# Pre-transplant weight loss predicts inferior outcome after allogeneic stem cell transplantation in patients with myelodysplastic syndrome

**DOI:** 10.18632/oncotarget.4805

**Published:** 2015-08-24

**Authors:** Aleksandar Radujkovic, Natalia Becker, Axel Benner, Olaf Penack, Uwe Platzbecker, Friedrich Stölzel, Martin Bornhäuser, Ute Hegenbart, Anthony D. Ho, Peter Dreger, Thomas Luft

**Affiliations:** ^1^ Department of Internal Medicine V, University Hospital Heidelberg, Heidelberg, Germany; ^2^ Division of Biostatistics, German Cancer Research Center, Heidelberg, Germany; ^3^ Campus Benjamin Franklin, Charité, Interdisziplinäre Klinik und Poliklinik für Stammzelltransplantation, Berlin, Germany; ^4^ Department of Internal Medicine I, University Hospital Carl Gustav Carus Dresden, Technical University Dresden, Dresden, Germany

**Keywords:** MDS, weight loss, allogeneic stem cell transplantation, relapse, outcome

## Abstract

Allogeneic stem cell transplantation (alloSCT) represents a curative therapeutic option for patients with myelodysplastic syndrome (MDS), but relapse and non-relapse mortality (NRM) limit treatment efficacy. Based on our previous observation in acute myeloid leukemia we investigated the impact of pre-transplant weight loss on post-transplant outcome in MDS patients. A total of 111 patients diagnosed with MDS according to WHO criteria transplanted between 2000 and 2012 in three different transplant centers were included into the analysis. Data on weight loss were collected from medical records prior to conditioning therapy and 3–6 months earlier. Patient, disease and transplant characteristics did not differ between patients with weight loss (2–5%, *n* = 17; > 5%, *n* = 17) and those without (*n* = 77). In a mixed effect model, weight loss was associated with higher risk MDS (*p* = 0.046). In multivariable analyses, pre-transplant weight loss exceeding 5% was associated with a higher incidence of relapse (*p* < 0.001) and NRM (*p* = 0.007). Pre-transplant weight loss of 2–5% and > 5% were independent predictors of worse disease-free (*p* = 0.023 and *p* < 0.001, respectively) and overall survival (*p* = 0.043 and *p* < 0.001, respectively). Our retrospective study suggests that MDS patients losing weight prior to alloSCT have an inferior outcome after transplantation. Prospective studies addressing pre-transplant nutritional interventions are highly warranted.

## INTRODUCTION

Therapeutic options for patients with high-risk myelodysplastic syndrome (MDS) have evolved over the past decade [1]. Specifically, hypomethylating agents (HMA) were demonstrated to prolong survival as compared to best supportive care in higher risk MDS patients, and the azanucleoside 5-azacitidine can currently be considered standard first-line therapy in this patient group [1, 2].

Despite these therapeutic advances, allogeneic stem cell transplantation (alloSCT) remains the only potentially curative treatment modality for patients with MDS, and transplant outcomes in MDS patients have substantially increased over recent years [1, 3, 4]. Depending on disease characteristics and patient age, long-term disease-free survival (DFS) can currently be achieved in around 30–40% of patients transplanted for MDS [4–6]. Most MDS patients are older than 60 years and post-transplant mortality remains a serious problem [7]. Particularly, age-related factors and comorbidities significantly affect the risk of non-relapse mortality (NRM) [8]. Therefore, only patients with higher risk MDS according to the International Prognostic Scoring System (IPSS) risk score (i.e. intermediate-2 or higher) [9] appear to profit from alloSCT [10, 11]. On the other side, advanced disease stage at transplantation has been shown to be associated with inferior survival after alloSCT with adverse cytogenetics and higher IPSS risk being major predictive factors of shorter DFS [5, 10, 12, 13].

We have recently provided evidence that weight loss and metabolic distress prior to alloSCT were associated with relapse and death risk in patients with acute myeloid leukemia (AML) in two independent patient cohorts [14]. In the present work, we investigated the influence of pre-transplant weight loss on clinical outcome after alloSCT in 111 patients with MDS according to WHO criteria using data from three different transplant centers.

## RESULTS

### Patient and transplant characteristics

A total of 111 patients were included into the analysis. Median age at time of alloSCT was 52 (range 19–72) years with 22% of patients being older than 60 years. AlloSCT was performed for the MDS WHO subtype RA(RS)/RCMD in 31 patients (28%), RAEB1 in 31 patients (28%) and RAEB2 in 49 patients (44%). The risk categories according to IPSS were intermediate-1 in 33 (34%), intermediate-2 in 44 (45%), high in 21 (21%), and information not available in 13 patients. Cytogenetic risk groups according to IPSS were favorable, intermediate and poor in 46%, 16% and 38% of the patients, respectively. The majority of the patients was previously untreated (*n* = 72, 65%). Nineteen (17%) and 14 patients (13%) received hypomethylating agents and AML-like chemotherapy prior to alloSCT, respectively. BM blast count directly prior to alloSCT was median 5% (range 0–19) and normal (<5%) in 43% of the patients.

Thirty-one patients (28%) received transplants from related donors (RD), 59 patients (53%) from matched unrelated donors (MUD) and 21 (19%) from mismatched unrelated donors (MMUD). The donor was female in 38% of the cases with 19 patients being sex mismatched with the donor. Ninety-three patients (84%) received reduced intensity conditioning (RIC) and 18 patients (16%) received standard myeloablative conditioning (MAC). Stem cell source was peripheral blood in 99 (89%) and bone marrow in 12 (11%) patients. Estimated median follow-up at the time of analysis of surviving patients was 36.1 months (95% CI 27.8–56.4; range 4.8–121.8). Patient and transplant characteristics are summarized in Table 1.

**Table 1:** Patient and treatment characteristics

	Whole cohort *n* = 111	Weight loss ≤2% *n* = 77	Weight loss 2–5% *n* = 17	Weight loss >5% *n* = 17	P*	P**
**Median age at alloSCT (years, range)**	52 (19–72)	53 (19–69)	50 (19–72)	51 (32–70)	0.88	0.30
**Age categories, *n* (%)**					0.75	0.75
<50 years	45 (41)	30 (39)	8 (47)	7 (41)		
50–60 years	42 (38)	31 (40)	4 (24)	7 (41)		
>60 years	24 (22)	16 (21)	5 (29)	3 (18)		
**Male gender, *n* (%)**	66 (59)	45 (58)	10 (59)	11 (65)	0.89	0.84
**MDS WHO subtype, *n* (%)**					0.82	0.60
RA(RS)/RCMD	31 (28)	24 (31)	4 (24)	3 (18)		
RAEB1	31 (28)	21 (27)	5 (29)	5 (29)		
RAEB2	49 (44)	32 (42)	8 (47)	9 (53)		
**IPSS risk, *n* (%)**					0.09	0.48
Intermediate-1	33 (34)	24 (36)	7 (44)	2 (13)		
Intermediate-2	44 (45)	31 (46)	7 (44)	6 (40)		
High	21 (21)	12 (18)	2 (13)	7 (47)		
Unknown	13	10	1	2		
**Cytogenetics, *n* (%)**					0.10	0.54
Favourable	49 (46)	32 (43)	12 (71)	5 (31)		
Intermediate	17 (16)	11 (15)	1 (6)	5 (31)		
Poor	41 (38)	31 (40)	4 (24)	6 (38)		
Unknown	4	3		1		
**Previous treatment, *n* (%)**					0.36	0.37
No	72 (65)	53 (69)	8 (47)	11 (65)		
HMA	19 (17)	13 (17)	4 (24)	2 (12)		
AML-like chemotherapy	14 (13)	7 (9)	3 (18)	4 (24)		
Immunosuppression	6 (5)	4 (5)	2 (12)	0 (0)		
**Donor, *n* (%)**					0.12	0.53
RD	31 (28)	23 (29)	2 (12)	6 (35)		
MUD	59 (53)	38 (49)	14 (82)	7 (41)		
MMUD	21 (19)	16 (21)	1 (6)	4 (24)		
**Stem cell source, *n* (%)**					0.53	1
PB	99 (89)	69 (90)	14 (82)	16 (94)		
BM	12 (11)	8 (10)	3 (18)	1 (6)		
**Sex match with the donor, *n* (%)**					0.16	0.20
Matched	67 (61)	45 (60)	12 (71)	10 (59)		
Recipient female – donor male	23 (21)	19 (25)	3 (18)	1 (6)		
Recipient male – donor female	19 (17)	11 (15)	2 (12)	6 (35)		
Unknown	2	2				
**Conditioning, *n* (%)**					0.27	0.58
RIC	93 (84)	66 (86)	12 (71)	15 (88)		
MAC	18 (16)	11 (14)	5 (29)	2 (12)		
**Median BM blast count prior to alloSCT (%, range)**	5 (0–19)	5 (0–19)	5 (1–15)	10 (3–19)	0.10	0.62
**BM blasts prior to alloSCT, *n* (%)**					0.34	0.79
<5%	42 (43)	30 (45)	8 (50)	4 (27)		
5–9%	22 (22)	14 (21)	5 (31)	3 (20)		
10–19%	34 (35)	23 (34)	3 (19)	8 (53)		
Unknown	13	10	1	2		
**Median BMI prior to alloSCT (kg/m^2^, range)**	25.1 (16.5–36.9)	26.3 (17.3–36.9)	24.3 (16.5–32.1)	24.1 (17.8–31.3)	0.11	0.37
**Period alloSCT performed (year)**	2000–2012	2000–2012	2000–2012	2001–2011	0.77	0.98

BM: bone marrow; BMI: body mass index; HMA: hypomethylating agent; MAC: myeloablative conditioning; MDS: myelodysplastic syndrome; MMUD: mismatched unrelated donor; MUD: matched unrelated donor; PB: peripheral blood; RD: related donor; RIC: reduced intensity conditioning; alloSCT: allogeneic stem cell transplantation.

* Patients with weight loss ≤2% *versus* patients with weight loss 2–5% *versus* patients with weight loss >5%.

** Patients with weight loss ≤2% *versus* patients with weight loss 2–5% and patients with weight loss >5%.

### Post-transplant outcome

**Figure 1:**
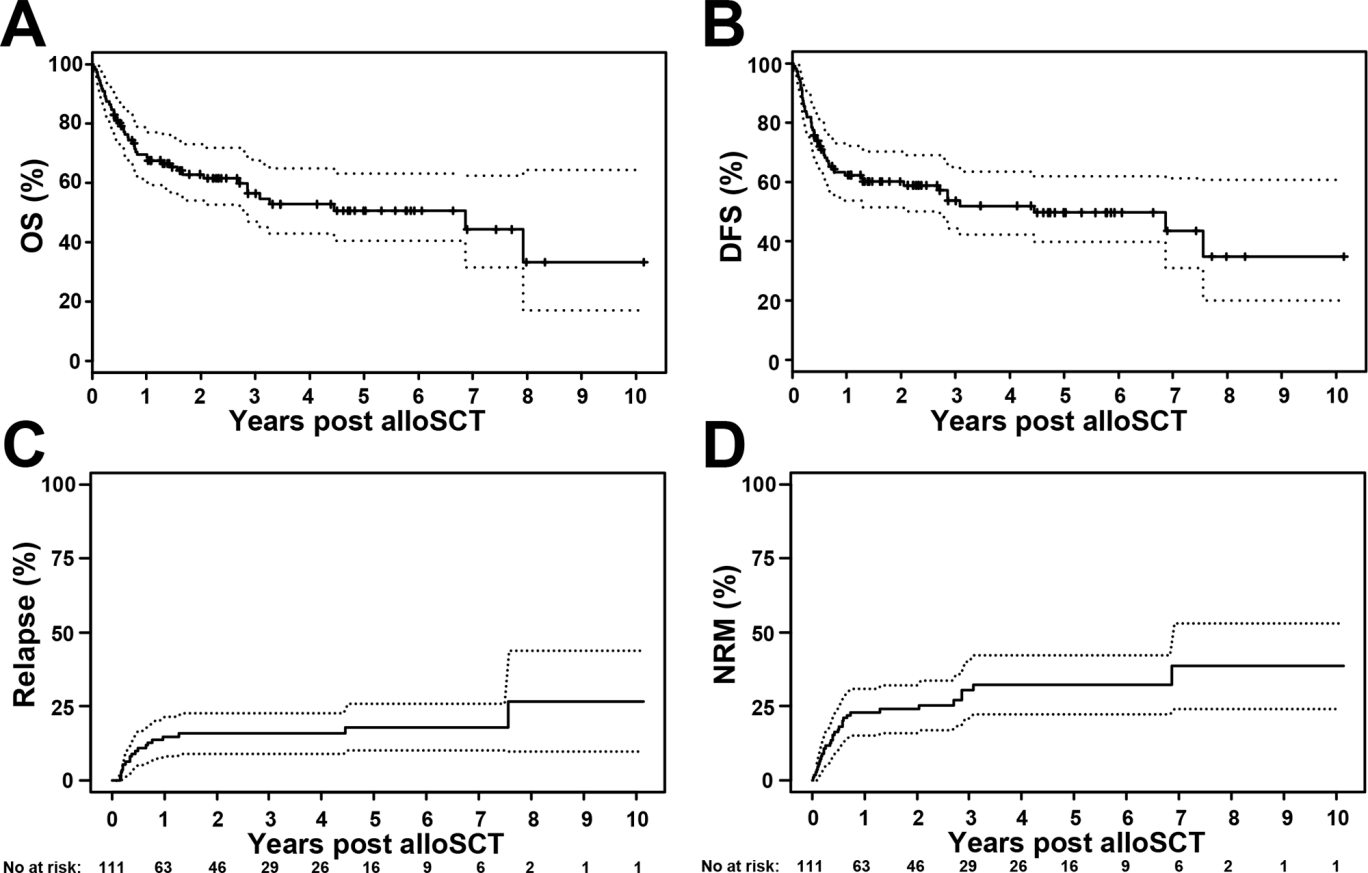
Overall survival (OS), disease-free survival (DFS) and cumulative incidences of relapse and non-relapse mortality (NRM) for the whole patient cohort (*n* = 111) **A.** For the entire cohort, the estimated probability of OS at 1 and 5 years after alloSCT was 70% (95% CI 61–79) and 51% (95% CI 41–63), respectively. **B.** The estimated probability of DFS at 1 and 5 years after alloSCT was 62% (95% CI 54–72) and 50% (95% CI 40–62), respectively. **C.** The cumulative incidence of relapse at 1 and 5 years after alloSCT was 15% (95% CI 8–21) and 18% (95% CI 10–26), respectively. **D.** The cumulative incidence of NRM at 1 and 5 years after alloSCT was 23% (95% CI 15–31) and 32% (95% CI 22–42), respectively. The dotted lines represent 95% confidence intervals.

A total of 48 (43%) patients had died by the time of analysis. Nineteen patients (17%) experienced MDS relapse at a median of 4.4 (range 1.9–90.7) months after alloSCT. A total of 32 patients (29%) died of transplant-associated causes at a median of 5 (range 0.2–82.4) months post alloSCT. For the entire patient cohort, estimated probability of survival was 70% (95% CI 61–79) and 51% (95% CI 41–63) at 1 and 5 years after alloSCT, respectively. The estimated probability of DFS was 62% (95% CI 54–72) and 50% (95% CI 40–62) at 1 and 5 years after alloSCT, respectively (Figure 1A and 1B). The cumulative incidence of relapse at 1 and 5 years after alloSCT was 15% (95% CI 8–21) and 18% (95% CI 10–26), respectively, and the cumulative incidence of NRM at 1 and 5 years after alloSCT was 23% (95% CI 15–31) and 32% (95% CI 22–42), respectively (Figure 1C and 1D).

### Prognostic factors for outcome: role of pre-transplant weight loss

A total of 34 (31%) patients experienced weight loss > 2% with 17 (15%) patients losing more than 5% weight in the period of 3–6 months prior to alloSCT (Table 1). Patient characteristics, disease and transplant characteristics did not differ between patients with weight loss and those without (Table 1). Moreover, it should be noted that patients who lost weight prior to alloSCT were not underweight: median body mass index (BMI) of patients with stable weight (weight loss < 2%) at alloSCT did not significantly differ from the BMI of patients experiencing weight loss (Table 1).

Univariate analyses showed that previous treatment with AML-like chemotherapy and transplantation from unrelated donors were associated with a significantly higher incidence of relapse and NRM (Table 2). Pre-transplant weight loss (both 2–5% and >5%) was associated with significantly increased relapse incidence and shorter DFS. Furthermore, pre-transplant weight loss exceeding 5% resulted in significantly higher NRM and, consequently, inferior overall survival after alloSCT. The results of the univariate analyses are given in Table 2.

**Table 2:** Prognostic factors of overall survival (OS), disease-free survival (DFS), relapse and non-relapse mortality (NRM) (univariate analysis)

	OS		DFS		Relapse		NRM	
	HR	*P*	HR	*P*	HR*	*P*	HR*	*P*
**Age at alloSCT (*n* = 111)**								
Per 10-year increase	1.29	0.080	1.24	0.114	1.25	0.245	1.31	0.192
**Recipient sex (*n* = 111)**								
Female	Ref		Ref		Ref		Ref	
Male	1.27	0.453	1.16	0.612	1.02	0.752	1.09	0.541
**MDS WHO subtype (*n* = 111)**								
RA(RS)/RCMD/RAEB1	Ref		Ref		Ref		Ref	
RAEB2	0.97	0.930	1.06	0.846	1.35	0.530	0.92	0.817
**IPSS risk (*n* = 98)**								
Intermediate-1	Ref		Ref		Ref		Ref	
Intermediate-2/high	1.72	0.138	1.95	0.060	2.38	0.182	1.78	0.180
**Cytogenetics (*n* = 107)**								
Favourable/intermediate	Ref		Ref		Ref		Ref	
Poor	1.63	0.114	1.39	0.268	1.66	0.315	1.26	0.533
**Previous treatment (*n* = 111)**								
No	Ref		Ref		Ref		Ref	
AML-like chemotherapy	1.01	0.983	1.33	0.511	**4.71**	**0.008**	0.45	0.278
HMA/IS	1.38	0.375	1.36	0.378	1.61	0.464	1.31	0.516
**Donor (*n* = 111)**								
UD	Ref		Ref		Ref		Ref	
RD	**0.45**	**0.027**	0.61	0.138	1.34	0.540	**0.30**	**0.024**
**Stem cell source (*n* = 111)**								
BM	Ref		Ref		Ref		Ref	
PB	1.03	0.947	1.20	0.685	2.43	0.397	0.95	0.924
**Conditioning (*n* = 111)**								
MAC	Ref		Ref		Ref		Ref	
RIC	1.05	0.901	1.18	0.656	1.89	0.407	0.98	0.964
**BM blast at alloSCT (*n* = 98)**								
≥5%	Ref		Ref		Ref		Ref	
<5%	0.93	0.838	0.84	0.602	0.82	0.726	0.86	0.696
**Pre-transplant weight loss (*n* = 111)**								
≤2%	Ref		Ref		Ref		Ref	
2–5%	1.91	0.101	**2.19**	**0.036**	**4.40**	**0.028**	1.66	0.279
>5	5.06	**<0.001**	**5.22**	**<0.001**	**14.19**	**<0.001**	**2.84**	**0.049**

BM: bone marrow; HMA: hypomethylating agent; HR: hazard ratio; IS: immunosuppression; MAC: myeloablative conditioning; MDS: myelodysplastic syndrome; MMUD: mismatched unrelated donor; MUD: matched unrelated donor; PB: peripheral blood; RD: related donor; RIC: reduced intensity conditioning; alloSCT: allogeneic stem cell transplantation; UD: unrelated donor.

* Cause-specific HR.

**Figure 2:**
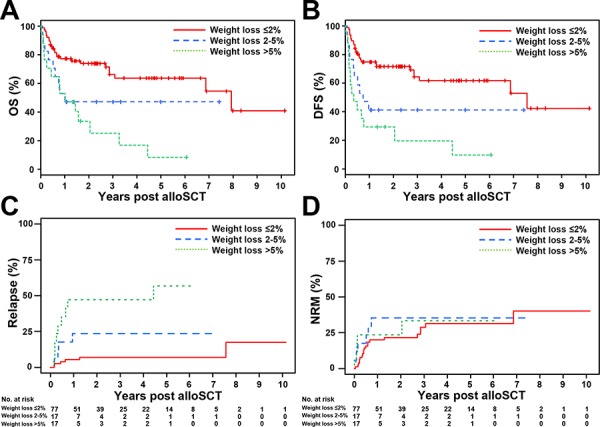
Overall survival (OS), disease-free survival (DFS) and cumulative incidences of relapse and non-relapse mortality (NRM) according to different weight loss categories **A.** For patients who did not lose weight, the estimated probability of OS at 5 years after alloSCT was 64% (95% CI 52–78). For patients who experienced weight loss of 2–5% and > 5% prior to alloSCT the estimated probability of OS was substantially lower reaching 47% (95% CI 28–78) and 8% (95% CI 1–53), respectively, at 5 years. **B.** The estimated probability of DFS at 5 years was 41% (95% CI 23–73) and 10% (95% CI 2–57) in patients displaying weight loss of 2–5% and > 5% prior to alloSCT, respectively, and lower as compared to MDS patients without weight loss (DFS at 5 years 62%, 95% CI 50–76). **C.** The cumulative incidences of relapse at 5 years were 24% (95% CI 3–44) and 57% (95% CI 31–82) in patients who lost weight (2–5% and > 5%, respectively) as compared to 7% (95% CI 1–13) in patients without weight lost. **D.** The cumulative incidence of NRM at 5 years in patients experiencing ≤ 2%, 2–5%, and > 5% weight loss prior to alloSCT were 31% (95% CI 19–44), 35% (95% CI 12–58), and 33% (95% CI 9–58), respectively.

Consequently, for patients who had experienced 2–5% and >5% weight loss prior to alloSCT, estimated probabilities of OS at 5 years after alloSCT were 47% (95% CI 28–78) and 8% (95% CI 1–53), respectively. Estimated probabilities of DFS at 5 years were 41% (95% CI 23–73) and 10% (95% CI 2–57), respectively, i.e. substantially lower as compared to MDS patients who did not experience weight loss (OS of 64%, 95% CI 52–78, and DFS 62%, 95% CI 50–76, at 5 years) (Figure 2A, 2B). Accordingly, cumulative incidences of relapse after 5 years were 24% (95% CI 3–44) and 57% (95% CI 31–82) in patients who lost weight (2–5% and >5%, respectively) as compared to 7% (95% CI 1–13) in patients without weight loss (Figure 2C). By comparison, incidence of NRM at 5 years was similar between the groups reaching 31% (95% CI 19–44), 35% (95% CI 13–58), and 33% (95% CI 9–58) in patients experiencing no, 2–5%, and >5% weight loss prior to alloSCT, respectively (Figure 2D).

In Cox regression analyses with relapse and NRM as endpoints, weight loss exceeding 5% prior to alloSCT was an independent predictor of relapse (*p* < 0.001) and NRM (*p* = 0.007) (Table 3). Both pre-transplant weight loss categories (2–5% and > 5%) along with donor source were independently associated with significantly inferior OS and DFS in multivariable analyses (Table 3).

**Table 3:** Prognostic effect of weight loss and covariates on overall survival (OS), disease-free survival (DFS) and the cumulative incidences of relapse and non-relapse mortality (NRM) (multivariable analysis*)

	OS		DFS		Relapse		NRM	
	HR (95% CI)	*P*	HR (95% CI)	*P*	HR^†^ (95% CI)	*P*	HR^†^ (95% CI)	*P*
**MDS WHO subtype**								
RA(RS)/RCMD/RAEB1	Ref		Ref		Ref		Ref	
RAEB2	0.75 (0.35–1.61)	0.459	0.72 (0.34–1.51)	0.380	0.54 (0.14–2.13)	0.381	0.73 (0.29–1.83)	0.502
**Previous treatment**								
No	Ref		Ref		Ref		Ref	
AML-like chemotherapy	0.33 (0.10–1.10)	0.071	0.52 (0.17–1.61)	0.257	4.70 (0.66–33.50)	0.122	0.16 (0.03–0.91)	**0.038**
HMA/IS	0.75 (0.32–1.78)	0.515	0.88 (0.38–2.03)	0.767	2.21 (0.42–11.82)	0.353	0.67 (0.25–1.80)	0.424
**IPSS risk**								
Intermediate-1	Ref		Ref		Ref		Ref	
intermediate-2/high	1.83 (0.79–4.26)	0.160	1.88 (0.84–4.20)	0.125	1.14 (0.25–5.28)	0.864	2.28 (0.84–6.18)	0.107
**Donor**								
UD	Ref		Ref		Ref		Ref	
RD	0.24 (0.10–0.60)	**0.002**	0.39 (0.17–0.87)	**0.022**	0.87 (0.25–2.95)	0.818	0.19 (0.06–0.66)	**0.009**
**Conditioning**								
MAC	Ref		Ref		Ref		Ref	
RIC	1.33 (0.58–3.04)	0.505	1.43 (0.63–3.24)	0.392	4.37 (0.76–25.29)	0.098	1.25 (0.47–3.31)	0.654
**BM blast at alloSCT**								
≥5%	Ref		Ref		Ref		Ref	
<5%	1.30 (0.63–2.68)	0.471	1.03 (0.51–2.10)	0.934	0.79 (0.23–2.70)	0.712	1.02 (0.44–2.37)	0.960
**Pre-transplant weight loss**								
≤2%	Ref		Ref		Ref		Ref	
2–5%	2.38 (1.03–5.51)	**0.043**	2.48 (1.13–5.42)	**0.023**	3.23 (0.80–13.08)	0.100	2.26 (0.83–6.18)	0.112
>5%	10.05 (3.84–26.30)	**<0.001**	7.53 (3.06–18.53)	**<0.001**	14.42 (3.21–64.86)	**<0.001**	5.96 (1.64–21.58)	**0.007**

HR: hazard ratio; IS: immunosuppression; MAC: myeloablative conditioning; RD: related donor; RIC: reduced intensity conditioning; UD: unrelated donor.

* Missing values imputed using “mice” function.

† Cause-specific HR.

### Predicting weight loss prior to alloSCT

In a mixed effect model with weight loss as endpoint, an association with higher risk MDS according to IPSS (*p* = 0.046) was observed, whereas age, recipient sex and previous treatment showed no significant interaction (Table 4).

**Table 4:** Mixed effect model with weight loss as endpoint and transplant center as random effect (linear regression)

	Estimate* ± SE	*P*
**Age at alloSCT**		
Per 10-year increase	0.06 ± 0.46	0.897
**IPSS risk**		
Intermediate-1	Ref	
Intermediate-2/high	2.46 ± 1.21	**0.046**
**Previous treatment**		
No	Ref	
AML-like chemotherapy	−0.70 ± 1.48	0.636
HMA/IS	0.88 ± 1.25	0.481
**Recipient sex**		
Female	Ref	
Male	0.06 ± 1.04	0.954

AML: acute myeloid leukemia; AlloSCT: allogeneic stem cell transplantation; HMA: hypomethylating agent; IS: immunosuppression; SE: standard error.

* Interpretation of the coefficients: For example, if the estimated coefficient for IPSS risk is 2.46, then the change from the IPSS risk category intermediate-1 to intermediate-2/high has a linear impact on weight loss (in %) by the factor 2.46.

## DISCUSSION

For patients with MDS, early mortality of alloSCT could be substantially reduced since introduction of reduced intensity conditioning (RIC), thereby making this potentially curative modality accessible for older patients who represent the bulk of patients at risk for MDS [11]. Nevertheless, there is still a considerable risk of late mortality mainly due to disease relapse that currently represents the major challenge in the post-transplant management [3, 4]. Recently, with the advent of high-throughput sequencing analyses and identification of recurrent somatic mutations, more and more nuanced risk stratifications predicting post-transplant outcome have become possible. Furthermore, several pharmacological and immunotherapeutic strategies to avoid relapse and increase treatment success after alloSCT were investigated [15]. However, no major advancement in reducing the risk of relapse after alloSCT has been made [16]. The present retrospective study introduces pre-transplant weight loss as a novel, strong predictor of relapse and worse survival after alloSCT for patients with MDS who were not transformed into AML. The present study extends and supports our previous report, in which weight loss and metabolic alterations prior to alloSCT were strongly associated with increased incidence of relapse and death in patients diagnosed with AML [14].

When considering the whole patient population, the estimated survival probabilities (OS and DFS) and cumulative incidences of relapse and NRM at 5 years in our study were generally comparable with literature data [5, 17, 18]. The predictive power of the IPSS risk categories regarding post-transplant outcome has been confirmed in several former studies [10, 13]. In our analyses, higher IPSS risk category prior to alloSCT showed a trend towards shorter DFS. In contrast, and at variance with previous reports [5, 19], both adverse cytogenetics and higher pre-transplant disease burden (BM blasts >5% at the time of alloSCT) were not significantly associated with post-alloSCT relapse and mortality. However, our study population comprised only patients who were not transformed into secondary AML. Similarly, in the study of Warlick et al. [17] reporting transplant outcomes for 84 patients with MDS including chronic myelomonocytic leukemia, the prognostic impact of cytogenetics and BM blast percentage at alloSCT on relapse incidence and DFS was considerably less pronounced. Furthermore, close to our study, stem cell source and type of conditioning did not influence outcome. However, in agreement with previous reports, unrelated donor transplantation resulted in higher cumulative incidences of NRM and inferior survival as compared with transplantation from related donors, whereas no difference in the incidence of relapse could be observed. [20, 21].

The most notable finding in the present study was certainly the identification of pre-transplant weight loss as a novel and possibly controllable risk factor that, in view of our previous report on AML patients [14], reproducibly predicted worse post-transplant outcome also in patients with MDS. For the older population, clinically significant weight loss is considered a decrease in weight exceeding 2%, 5%, or 10% of baseline body weight in 1, 3, or 6 months, respectively [22]. For cancer patients, loss of 5% body weight over past 6 months represents one possible definition of cachexia [23]. Weight loss and malnutrition are long-established risk factors of poor prognosis in patients diagnosed with different malignancies [24]. In the present study, patients who had lost more than 5% of their body weight over the previous 3–6 months prior to alloSCT had a more than 10-fold higher risk of death as compared to those who maintained their weight. Weight loss was an independent risk factor of poor survival as a result of both increased risk of relapse and NRM as revealed by multivariable analyses. Interestingly in our patients, pre-transplant weight loss appeared to be associated only with higher risk MDS (intermediate-2/high according to IPSS), but not with age or previous treatment. In addition, as yet undefined disease or patient dependent factors may also be involved.

The impact of patient weight on outcome after alloSCT was analyzed in several previous studies suggesting an increased risk of death in the early post-transplant period and decreased overall and relapse-free survival for underweight patients [25, 26]. Furthermore, in a recently published large registry study, Fuji et al. [27] could show that underweight (pre-transplant BMI < 18.5 kg/m^2^) was independently associated with poor survival because of a significantly increased risk of relapse, whereas obesity (BMI ≥ 30 kg/m^2^) predicted higher NRM. However, all the aforementioned studies neither assessed the change of body weight before alloSCT nor investigated its impact on post-transplant outcome. The present study together with our previous report on AML patients [14] suggests that weight loss before alloSCT, rather than a lean body mass itself, may constitute a highly relevant factor influencing outcome after alloSCT, especially in view of the fact that MDS patients with pre-transplant weight loss, similar to AML, were not underweight (median BMI > 24 kg/m^2^).

It should be noted though that MDS patients differ from AML patients with regard to the heterogeneity of their treatment prior to alloSCT. Whilst all AML patients received at least one cycle of induction therapy before transplant, MDS patients may be untreated for several years (up-front transplantation), or induced with AML-like chemotherapy, or received alternative or experimental induction therapies (HMA, immunosuppression etc.). Consequently, it was not possible to evaluate weight loss from initial diagnosis as in AML. Instead, changes in body weight over the previous 3–6 months prior to alloSCT preceding transplantation were collected and analyzed in MDS patients. The impact on survival in our study is surprisingly strong. However, besides the association with higher risk disease in our study, the pathophysiology of pre-transplant weight loss and its implications in the context of alloSCT and MDS remain uncertain.

In studies on hematopoietic and non-hematopoietic stem cells, energy deprivation was shown to induce exit from cell cycle [28–30]. Therefore, weight loss and energy deprivation might induce and promote quiescence of leukemic cells resulting in reduced sensitivity to chemotherapy and enhanced disease recurrence. On the other hand, nutrition and patient's nutritional status have been shown to influence the pharmacokinetics of drugs and chemotherapeutic agents [31, 32]. Consequently, alterations of the nutritional homeostasis prior to alloSCT may change the metabolism of anti-neoplastic agents and, accordingly, alter the efficacy of the conditioning regimen and/or the immunosuppression. This may then result in higher rates of disease recurrence and NRM and shorter post-transplant survival. Regardless of these hypotheses, pre-transplant weight loss represents a risk factor that can potentially be influenced, and prevention of body weight loss prior to alloSCT by nutritional support and/or lifestyle modification might constitute a feasible approach to improve the outcome of MDS patients.

We are aware that there are potential sources of bias in our analysis which are inherent to the retrospective nature of the study. Factors to consider include: sample size, patient selection, missing data in a proportion of patients, and different types of pre-transplant treatment and conditioning. Moreover, it is obvious that further validation of our results is required and that the impact of nutritional status and body weight change on post-transplant outcome can only be assessed in a prospective interventional study. However, since treatment opportunities in MDS patients relapsing after alloSCT are very limited [16], our findings strongly advocate such clinical studies and thus may help to improve the outcome and the clinical management of MDS patients undergoing alloSCT.

In conclusion, within the limitations of this study we demonstrate that weight loss prior to alloSCT was significantly associated with inferior outcome. Prospective interventional studies to assess the impact of pre-transplant nutritional support are highly warranted.

## PATIENTS AND METHODS

### Patients and eligibility criteria

Patients diagnosed with MDS according to WHO criteria [33] who underwent alloSCT between 2000 and 2012 in three different transplant centers (Heidelberg, Dresden and Berlin) were identified reviewing electronically filed patients charts. As secondary AML patients were already part of our previous report [14], only MDS patients with less than 20% BM blasts (relating to lifetime maximum BM blast count prior to alloSCT) were included into this analysis. Written informed consent for participation in the retrospective study was obtained according to the declaration of Helsinki in all eligible patients, and the local Ethics committees had approved data collection.

### Definitions

Disease stage and cytogenetic risk were assessed according to IPSS [9]. Relapse was defined as hematologic recurrence of MDS according to published standardized criteria [34]. Reduced intensity conditioning (RIC) was defined as use of fludarabine associated with ≤8 Gy total-body irradiation (TBI), or busulfan ≤8 mg/kg, or other nonmyeloablative drugs. Myeloablative conditioning (MAC) regimens included TBI 12 Gy or busulfan more than 8 mg/kg in combination with other cytotoxic drugs. A matched unrelated donor (MUD) was defined as a donor-recipient pair matched for 10 of 10 HLA antigens using high-resolution molecular typing for the HLA-A, -B, -C, -DRB1, and -DQB1 genes.

### Statistical analysis

Weight data were retrospectively collected by medical chart review by three independent researchers in three transplant centers (Heidelberg, Dresden and Berlin). Weight loss was calculated on the basis of recorded weight data at the time of alloSCT (prior to the start of the conditioning treatment) and the maximum weight in the period of 3–6 months preceding the alloSCT. The cut-off values for weight loss (2% and 5%) were chosen because they represent possible definitions of cancer cachexia in a clinical setting [23]. Categorical and non-categorical data of patient and treatment characteristics between the patient cohorts were compared using Fisher's exact or the χ^2^ test and the Mann-Whitney or the Kruskall Wallis test, respectively.

Distributions of survival times were estimated by using the method of Kaplan and Meier. The confidence interval (CI) estimation was performed using Greenwood's formula for the variance of the survival function. The follow-up times were calculated by the reverse Kaplan-Meier estimate [35]. Overall survival (OS), DFS, incidence of relapse and NRM were calculated from the date of alloSCT to the appropriate endpoint. Cox regression analysis was applied for OS, DFS, relapse and NRM. Relapse and NRM were considered as competing risk events. Prognostic impact of weight loss on OS, DFS, relapse incidence and NRM was evaluated on the basis of hazard ratios (HR) with 95% CI from Cox's proportional hazards regression model with transplant center as a stratification term. The proportional hazards assumption was tested as proposed by Grambsch and Therneau [36]. Multivariable cause-specific Cox (proportional hazards) regression models were used to adjust effects for additional covariates: WHO grade, IPSS risk score, previous treatment, donor, conditioning and BM blast count at alloSCT. In case of competing events, cumulative incidences of relapse and NRM were estimated using the Aalen-Johansen estimator [37]. The influence of MDS-associated risk variables on weight loss was evaluated using a linear mixed effects model with transplant center as random effect. Incomplete multivariable data were imputed by chained equations computing 20 imputed data sets [38].

Calculations were done using the statistical software environment R, version 3.0.1, together with the R packages ‘coin’, version 1.0–23, ‘rms’, version 4.2–0, cmprsk, version 2.2–7, kmi, version 0.5. All statistical tests were two-sided. HR were estimated with 95% CI. Values of *P* <.05 were considered statistically significant.
